# Functional and Transcriptional Downregulation of Glutamate Transporters (EAAT1 and EAAT2) *via* DNMT3B Overexpression in Glial Cells

**DOI:** 10.1007/s12035-026-06070-w

**Published:** 2026-07-24

**Authors:** Jaqueline Loaeza-Loaeza, Ada G. Rodríguez-Campuzano, Luisa C. Hernández-Kelly, Daniel Hernández-Sotelo, Marie-Paule Felder-Schmittbuhl, Arturo Ortega

**Affiliations:** 1 Departamento de Toxicología, Centro de Investigación y de Estudios Avanzados del Instituto Politécnico Nacional, 07360 Mexico City, Mexico; 2 Facultad de Ciencias Químico-Biológicas, Universidad Autónoma de Guerrero, 39070 Chilpancingo, Guerrero Mexico; 3 Centre National de la Recherche Scientifique, Université de Strasbourg, 67000 Strasbourg, France; 4 Secretaría de Investigación, Ciencia, Humanidades, Tecnología e Innovación (SECIHTI), 03940 Mexico City, Mexico

**Keywords:** Glial glutamate transporters, DNA methylation, Glial cells, DNMT3B

## Abstract

Glutamate (Glu) is the major excitatory neurotransmitter amino acid in the vertebrate brain. It elicits its actions through the activation of specific receptors in neurons and glial cells. Excitatory amino acid transporters 1 and 2 are the Glu transporters expressed in glial cells, responsible for recycling Glu and executing a protective role against excitotoxicity and neurodegeneration. In neurogenerative diseases and gliomas, glial cells fail to maintain Glu-glutamine homeostasis, triggering excitotoxicity and DNA methylation profile disruption. The aim of this contribution was to investigate the relationship between the methylation gained by DNA methylase 3B overexpression and the functional effect and the expression levels of Glu transporters in glial cell models. First, we confirmed the differential expression and deregulation of excitatory amino acid transporters 1 and 2 in cancer and neurodegenerative diseases. Next, Müller retinal cells were transfected with a DNA methylase 3B vector, and global 5-methylcytosine increased was detected after 48 h. Using a [^3^H]-D-Aspartate uptake assay in Müller glia cells and the tumoral U87 cells, we determined that de novo methylation affects the functional activity of the Glu transporters. The overexpression of DNMT3B also decreases the expression of Glu transporters in both cell models. Finally, due to the structural characteristics related to methylation deposition in DNA, we analyzed the methylation status of the excitatory amino acid 2 promoter, and we confirmed that DNA methylation increased in U87 cells by the DNMT3B overexpression. These findings suggest that de novo DNA methylation regulates the expression of glial Glu transporters and affects their functional role under excitotoxic environments. This work highlights the DNA methylation mechanism as a promising approach to understand the physiopathology landscape related to excitotoxic processes involved in brain diseases.

**Supplementary Information:**

The online version contains supplementary material available at 10.1007/s12035-026-06070-w.

## Introduction

Glutamate (Glu) is the major excitatory amino acid neurotransmitter in the central nervous system (CNS) involved in 90% of all synapses to perform higher-order functions in humans [1]. Once released, Glu binds to specific membrane receptors, triggering diverse signaling cascades that shape brain function in the immediate, mediate, and long term. Glu removal from the synaptic cleft is carried out almost exclusively by a family of sodium-dependent excitatory amino acid transporters 1–5 (EAAT1-5) present in glial cells. EAAT1, also known as Glu/aspartate transporter (GLAST), is abundant in cerebellar Bergmann glial cells and retinal Müller glial cells. EAAT2, named in rodents glutamate transporter 1 (GLT1), is enriched in astrocytes and less expressed in oligodendrocytes, microglia, and in some neurons [2]. The dysregulation of Glu transporters leads to inefficient clearance of the amino acid from the extracellular space, and the prolonged activation of the extra synaptic N-methyl-D-aspartate (NMDA) receptors that eventually triggers a neuronal death cascade in the process known as excitotoxicity [3, 4]. The homeostatic expression of Glu transporters has been regarded as a protective mechanism that avoids excitotoxicity [5, 6].

The disruption of proper glial cell function in neurodegenerative pathways and gliomas critically accelerates downstream neuronal cell death. Aberrant expression of EAAT1 and EAAT2 is prevalent in some neurodegenerative diseases like Alzheimer’s disease (AD) [7, 8], Parkinson’s disease (PD), amyotrophic lateral sclerosis (ALS), Huntington’s disease (HD) [9], and malignant gliomas [10].

Brain cells have conserved gene spatiotemporal expression profiles that define their identities and functions through tight genetic and epigenetic control [11, 12]. The transcriptome profile in the brain is highly regulated by epigenomic modification of DNA, RNA, and proteins [13]. In the adult human brain, ~80% of CG dinucleotide and 1.5% of non-CG (CH, where H = A, T, or C) sites are methylated [14]. Specifically, a strong relationship exists between transcripts and DNA methylation profiles in development, learning, memory, and disease in the human CNS [15]. DNA methylation is a chemical modification that responds to immediate stimulus, established and maintained by the DNA methyltransferase enzymes (DNMTs). This chemical modification in promoter regions regulates the repressive transcription status of several genes in the diseased CNS that are related to excitotoxicity by high Glu levels. In this context, aging is the best characterized risk factor for the most prevalent ND and is strongly accompanied by an accelerated epigenetic imbalance evaluable through CpG sites and histone methylation [16, 17]. Altered methylation has been associated with neurodegenerative disease such as AD and PD, where aberrantly methylated genes have a significant contribution to these pathologies [18, 19]. A deregulation of the methylation machinery occurs in several disorders related to excitotoxic cell death, which is correlated with de novo DNA methyltransferases, such as DNMT3B. Physiological studies have demonstrated that normal DNMT3B expression levels are essential to avoid neuropathology and cognitive impairment in AD mouse models exposed to chronic hypoxic conditions [20]. In cerebrovascular accidents, the RNA-binding protein ELAV1 is bound to DNMT3B; this complex allows the PTEN-induced putative kinase 1 (PINK1) gene to remain unmethylated, resulting in ferroptosis attenuation [21]. Accordingly, de novo methylation has been reported as a frequent process in oxidative DNA damage conditions directed by the coordination between DNA polymerase β and DNMT3B [22]. All characterized examples have a common excitotoxic environment regulated by some key genes that are under methylation control. In the most aggressive and common form of primary brain cancer, glioblastoma (GBM), methylation is a key transcriptional regulator. These tumor cells feature a Glu release/uptake deregulation, and hypermethylation of metabolic genes, like enolase 1 (ENO1), glyceraldehyde-3-phosphate dehydrogenase (GAPDH), hexokinase 3 (HK3), and lactate dehydrogenase (LDHA) has been demonstrated [23].

A profound understanding of gene regulatory mechanisms is pivotal for the formulation of novel therapeutics and prevention strategies for some disorders related to the Glu excitotoxic process and DNA methylation, in which, glial cells are critically involved. This is a preliminary study that specifically explores key genes deregulated in glial cells by Glu excitotoxic levels and a plausible DNMT3B-mediated de novo methylation regulatory axis. Emphasis has been placed in the main excitotoxity regulators in glial cells, *SLC1A3* (EAAT1) and *SLC1A2* (EAAT2), and the key elements that regulate their transcription rates. In A172 cells, EAAT2 regulation by its CpG island methylation was shown for the first time [24]. Later, deep molecular exploration revealed key functional elements presented in the EEAT1 and EAAT2 promoters. Experimental evidence shows a strong transcriptional control by nuclear factor kappa-light chain-enhancer of activated B cells (NF-Kβ) that increases glial Glu transporters expression; in contrast, Ying-Yang 1 (YY1) represses these genes [25–27]. There are a few insights into the additional mechanisms that coordinate this fine regulation, for example, the repressor functions of YY1 are related to epigenetic repressors like the *Polycomb* proteins and histone deacetylases, which in turn are co-regulators of the DNMTs [28, 29]. This context is particularly relevant for *SLC1A2*, which presents epigenetic *cis*-regulatory elements like a large CpG island on its 5′ end, proven to be important for methylation deposition in its promoter regions. The methylation functional effect is genomic region-dependent; the readers for this chemical modification switch on and off gene transcriptional states. In promoters, the DNA methylation is associated with other repressive transcriptional marks on histones to silent larges genomic sequences, but it is equally important that the single cytosine methylation, which can regulate the binding of key transcription factors and thus regulate gene expression [30].

In our research group, we have recently evaluated the role of DNA methylation in the function of glial Glu transporters in primary cultures of chick cerebellar Bergmann glial cells. Demethylating treatments result in an augmentation of the Glu uptake, while the treatment with Glu decreases the uptake and increases the DNMT3B expression [31]. These results confirmed the possible relevance of methylation in the functional regulation of transporters through DNMT3B and provided the basis for the present work.

In the present study, we concentrate on glial Glu transporters in two lines cells models, focusing on their function and their regulation by the de novo DNA methyltransferase, DNMT3B. We aim to gain insight into a possible excitotoxic glial axis that involves de novo methylation and glial Glu transporters deregulation. Using cultured glial cell models with DNMT3B overexpression, we document here an increase in global 5mC levels, followed by a decrease in Glu uptake activity. Our data reveals a diminution of *SLC1A2* and *SLC1A3* mRNA levels in U87 and MIO-M1 cells. Accordingly, de novo methylating conditions driven by DNMT3B overexpression result in the gain of methylation in key regions of the *SLC1A2* promoter. Our results support the notion of the importance of dynamic DNA methylation in the excitotoxic process related to deficient glial Glu removal and the disturbance of key neuroprotective roles of glial cells.

## Material and Methods

### Materials

L-Glu was obtained from Tocris Biosciences (Ellisville, IL, USA). D-Aspartic acid (A8881) and all other chemicals were purchased from Sigma-Aldrich (St. Louis, MO, USA). The Bradford reagent was obtained from Bio-Rad (Hercules, CA, USA). [^3^H]-D-Aspartate was purchased from PerkinElmer (Boston, MA). Cell culture medium was from Thermo Fisher Scientific (Carlsbad, CA), and plasticware was purchased from Corning (New York, NY).

### Bioinformatic Data Processing

We used GEPIA, an interactive tool for analyzing the RNA-seq expression data of 9736 tumors and 8587 normal samples from The Cancer Genome Atlas (TCGA) and the Genotype-Tissue Expression (GTEx) projects. In our study, we used tumor/normal samples data for differential expression analysis using RNA-seq assessed via GEPIA (http://gepia.cancer-pku.cn/). The median values were downloaded and compared for *SLC1A2* and *SLC1A3* genes for distinct types of cancers: adrenocortical carcinoma (ACC), breast invasive carcinoma (BRCA), cholangio carcinoma (CHOL), lymphoid neoplasm diffuse large B-cell lymphoma (DLBC), glioblastoma multiforme (GBM), kidney chromophobe (KICH), kidney renal clear cell carcinoma (KIRC), kidney renal papillary cell carcinoma (KIRP), brain lower grade glioma (LGG), lung adenocarcinoma (LUAD), ovarian serous cystadenocarcinoma (OV), pheochromocytoma and paraganglioma (PCPG), rectum adenocarcinoma (READ), skin cutaneous melanoma (SKCM), testicular germ cell tumors (TGCT), thymoma (THYM), and uterine carcinosarcoma (UCS). Given the normal functionality of these transporters and the nature of this study, we focused on LGG and GBM. ANOVA test was conducted to determine statistical significance between normal and tumor samples.

For the proteomics profile in neurodegenerative diseases, datasets were obtained from the European Molecular Biology Laboratory – European Bioinformatics Institute (EMBL-EBI) data resources through the online tool Expression Atlas (https://www.ebi.ac.uk/gxa/home). The heatmaps generated for SLC1A2 and SLC1A3 correspond to the mean expression of the replicates for each disease that are part of the publication: [32].

### Cell Culture and Transfections

The immortalized human retina Müller glia cell line (MIO-M1) was from the Institute of Ophthalmology, University College, London, UK, while the U87MG cell line was purchased from the American Type Culture Collection (ATCC, USA). MIO-M1 cells were grown in DMEM/F12 1: 1 medium (Sigma-Aldrich) supplemented with 10% fetal bovine serum (FBS, PAA Laboratories GmbH, Austria), 100 U/mL of penicillin (Penprocilin 800,000 U, Lakeside, Mexico), and 100 μg/mL of streptomycin (Sulfestrep, Pisa Laboratories, Mexico). U-87MG cells were cultured in Dulbecco’s modified Eagle medium (DMEM-F12 HAM), supplemented with 10% fetal bovine serum FBS and 1% of antibiotic solution. The cells were grown at 37 °C in presence of 5% CO_2_. MIO-M1 or U87 were seeded in 6-well plates and transfected with 1.5 or 2 µg of the construction pCDNA3.1-DNMT3B generated by Peralta-Arrieta et al. [33], and for the excitotoxic controls, 1 mM of L-Glu or D-Aspartate were used. The transfections were made using lipofectamine 2000 Reagent (Lipofectamine, 2000 Invitrogen, Life Technologies, Carlsbad, CA, USA). After 48 h post-transfection or treatments, the cells were harvested for RNA (Direct-Zol RNA miniprep, ZymoResearch) and DNA (Wizard® Genomic DNA Purification, Promega Madison, USA) extraction.

### RNA Extraction and RT-qPCR Assay

Total RNA was extracted with the TRIzol reagent (Invitrogen; Carlsbad, CA, USA) according to the manufacturer’s protocol. The RNA concentrations were determined by spectrophotometry with a Nano-Drop 2000c spectrophotometer (Thermo Fisher Scientific, Waltham, MA, USA). RT-qPCR was performed with the KAPA SYBR FAST One-Step (Kapa Biosystems) in a reaction volume of 10 μL (50 ng of total RNA, dNTP (10 nM), Rox reference dye (1×), Kapa RT mix (1×) Kappa SYBR master mix, forward primer (200 nM), and reverse primer (200 nM)). The reaction was performed in ABI Step One Plus Real-Time PCR System (Applied Biosystems). The conditions of reverse transcription and amplification were 42 °C for 5 min followed by inactivation of the RT at 95 °C for 5 min and then 40 cycles at 5 s at 95 °C, 30 s at 60 °C, and 30 s at 72 °C. Melting curves were constructed to determine the purity and to verify whether the bands corresponded with theoretical melting temperatures. Data were normalized with RNA18SN1 as an internal control, and the relative expression was calculated using the 2−(^ΔΔ^Ct) method. Primer sequences were as follows: DNMT3B forward ACCACCTGC TGAATTACTCACG, DNMT3B reverse GATGGCATCAATCATCACTGG; GLT1 forward AACAATATGCCCAAGCAGGT, GLT1 reverse CTCCCAGGATGACACCAAAC; GLAST1 forward TACGAGTGACAGCTGCAGAT, GLAST1 reverse TCCATGGCCTCAGACACATT; and RNA18SN1 forward AGTCCCTGCCCTTTGTACACA, RNA18SN1 reverse GATCCGAGGGCCTCACTAAAC.

### Genomic DNA Isolation and DNA Methylation Quantification

Genomic DNA (gDNA) was extracted using the Wizard Genomic DNA Purification Kit (Promega; Madison, WI, USA) according to the instructions provided by the manufacturer. DNA quality and concentration were evaluated using an Epoch Spectrophotometer (Bio-Tek, USA) and through its integrity analysis in 1.5% agarose gels. The DNA Colorimetric Quantification Kit (ab117128) (Abcam, Cambridge, UK) was used to determine global DNA methylation of glial cells according to the manufacturer’s instructions. Briefly, a binding buffer was added to each well; then, a negative control, a positive control, or 100 ng of gDNA per reaction was added. The plate was incubated for 90 min, washed, and incubated with the capture antibody for 60 min. The plate was washed and incubated with the detection antibody after which an enhancer solution was added. Finally, the plate was washed and developing solution was added followed by stop solution. Absorbance was determined at 450 nm. The relative methylation status was calculated according to manufacturer’s protocol.

### Methylation-Specific PCR (MSP)

After DNA genomic isolation using the Wizard Genomic DNA Purification Kit (Promega; Madison, WI, USA), 1.5 or 2 μg of DNA of U87 cells transfected with con pCDNA3.1-DNMT3B or pCDNA3.1(+) by 48 h were treated with an EZ DNA Methylation-Gold™ kit (Zymo Research, Irvine, CA, USA). Briefly, 2 μg of genomic DNA was modified for each sample and MSP analysis was performed in a total of 10 μL, containing 1 μL of bisulfite-treated DNA, 250 nM of each primer, and AmpliTaq Gold360 Master Mix (Applied Biosystems, Foster City, CA, USA). The amplification conditions were as follows: denaturation, 95 °C for 10 min; 30 to 35 cycles of amplification, 30 s at 95 °C, 30 s at 60 °C, and 30 s at 72 °C; and a final extension of 72 °C for 10 min. Primer sequences were as follows: *SLC1A2* Pair 1 forward GATCGCGCGGAGATTCGGGATG, *SLC1A2* Pair 1 reverse ACCGCTCGACTCCGCTAAACCT; and *SLC1A2* Pair 2 forward GGGATTGTAAGGTTTAGTTTCGTC, *SLC1A2* Pair 2 reverse GACGATTAAAAAAATTACCCGAA.

### [^3^H]-D-Aspartate Uptake

Transfected cells were plated in 24-well plates (5 × 10^5^ cells/well) and cultured at 37 °C for 48 h after treatments. Next, the [^3^H]-D-aspartate uptake assay was carried out as previously described [34]. Once the treatment periods finished, cultured monolayers were washed twice with pre-warmed uptake buffer (HEPES-buffered solution containing 25 mM HEPES, 130 mM NaCl, 5.4 mM KCl, 1.8 mM CaCl_2_, 0.8 mM MgCl_2_, 33.3 mM glucose, and 1 mM NaH_2_PO_4_, pH 7.4) and were incubated with fresh pre-warmed uptake buffer containing 0.4 μCi/mL [^3^H]-D-aspartate ([^3^H]-D-Asp) (specific activity: 16.5 Ci/mmol, Perkin Elmer, MA, USA). D-Asp (Glu analog) was used as it has the advantage of being non-metabolizable while still being taken up by the same transporter systems as Glu. The uptake was finished after 30 min of incubation by the addition of ice-cold uptake buffer, and cells were lysed with 0.1 N NaOH. Protein concentration in the lysates was determined with Bradford protein assay (Bio-Rad, CA, USA) and then transferred to scintillation vials. Radioactivity was measured in a PerkinElmer Tri-Carb 2810TR liquid scintillation counter (PerkinElmer, MA, USA). Experiments were performed in quadruplicate in three to four independent cultures for MIO-M1 and U87 cells.

### Statistical Analysis

The results were analyzed with the GraphPad Prism Software (La Jolla, CA, USA). A one-way or two-way analysis of variance was carried out to determine significant differences between conditions. When these analyses indicated significance, Dunnett’s, Bonferroni’s, or Dunn’s post hoc test was used. All the results are presented as mean ± standard deviation (SD). In all cases, a *p*-value less than 0.05 was considered statistically significant.

## Results

### Glu Transporters are Differentially Expressed in CNS Pathological Processes

**Fig. 1:**
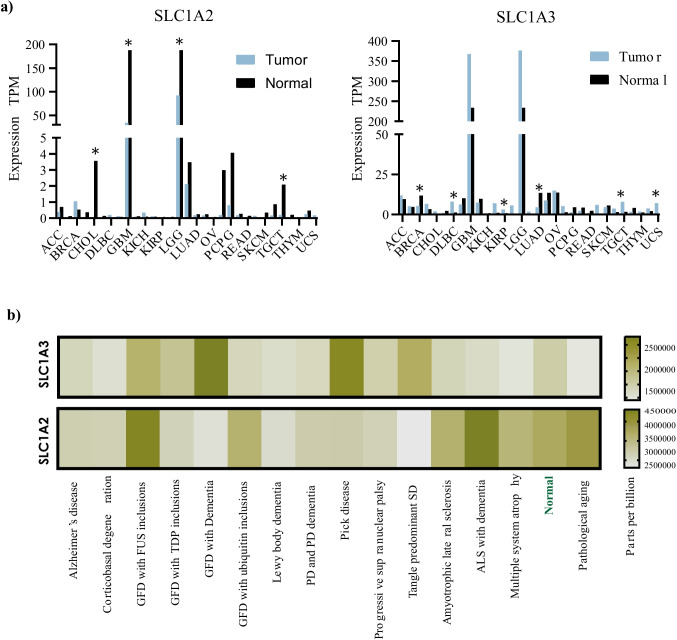
*SLC1A2* and *SLC1A3* are deregulated in CNS diseases. **a** TCGA dataset shown high level expression of *SLC1A2* and *SLC1A3* in GBM (glioblastoma) and LGG (low-grade gliomas) respect other tissues. Furthermore, in GBM and LGG, we found a Glu transporters transcripts differential expression between normal and tumor tissues with significant differences in *SLC1A2* (*). The expression media between tumor and paired normal samples is shown as transcripts per million (TPM). **b **Data from expression atlas EMBL-EBI were downloaded (Johnson ECB, Dammer EB, Duong DM, Ping L, Zhou M, et al., 2020), and we show the differential expression levels of Glu transporters in prevalent neurodegenerative diseases. All samples are from dorsolateral prefrontal cortex tissue derived from proteomic analysis with different numbers of replicates and age ranges; data are available in Expression Atlas

Glu extracellular levels are regulated by the steady-state balance between release and uptake. EAAT1/GLAST1 and EAAT2/GLT-1 are the main plasma membrane Glu transporters in glial cells responsible for carrying out the efficient synaptic Glu removal to avoid excitotoxic neuronal death, a hallmark of several neurological disorders and CNS tumors. These transporters are mainly regulated by post-transcriptional processes, albeit transcriptional regulation by several molecular elements, such as specific transcription factors, alternative transcription start sites, epigenetic marks, and polymorphisms are also present [35]. In this work, we gathered information on cancer from TCGA datasets and found significant changes in the expression of *SLC1A2* and *SLC1A3* (Fig. 1a) in several tumors. We focus on glioblastoma multiforme (GBM) and low-grade gliomas (LGG), two frequent CNS tumors of glial cells. This database revealed significant changes in the decreased expression level of *SLC1A2* in both cancer types when compared to normal tissue samples. *SLC1A3* also showed differences, but these did not reach statistical significance; however, we decided to include it in this exploration because, up to this stage, the correlation with methylation has not been evaluated. Screening *SLC1A2* and *SLC1A3* expression in prevalent neurodegenerative diseases is key because excitotoxicity from the improper functioning of glial cells is common in these disorders. For this exploration, we did not find a dataset that included expression data for representative NDs, so we decided to use a proteomic dataset available in the EMBL-EBI database. These proteomic data were processed from *postmortem *dorsolateral prefrontal cortex tissue samples, and it gives away a deregulated Glu transporters landscape in a patient cohort. The comparison changes to normal tissue; we have found that it is common for *SLC1A2* and *SLC1A3* expression to be deregulated (up and downregulation) in these diseases (Fig. 1b).

### DNMT3B Overexpression Increases DNA Global Methylation and Decreases Glu Uptake

**Fig. 2:**
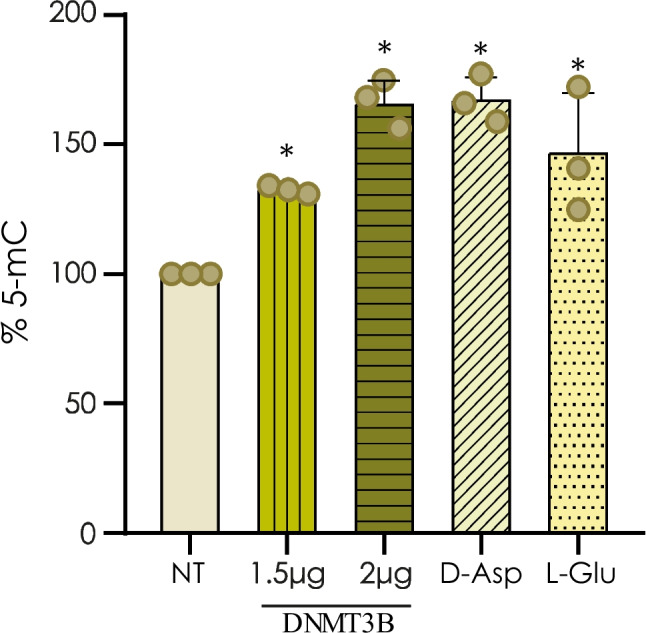
5mC levels increases after DNMT3B overexpression in Müller retina cells. Global DNA methylation was measured in MIO-M1 cells with DNMT3B overexpression for 48 h. Graph present changes in the percentage of methylated cytosine compared between treated vs non-treated cells (NT). MIO-M1 transfected with 1.5 µg of DNMT3B plasmid vector (1.5 µg) shown an increased although it is not statistically significant. Cells transfected with 2 µg of DNMT3B plasmid vector (2 µg) increase significantly 5-mC levels, suggesting a dose-dependent increase in de novo methylation. The excitotoxic condition were induced with 1 mM D-Asp (D-Asp) or 1 mM L-Glu (L-Glu), show a 5-mC increase in MIO M1 cells. Values represent mean ± standard deviation (SD) of three independent experiments. A one-way ANOVA was performed to determine whether there were significant differences between treated groups and the control with a Dunnett test (Prism 9 software) *p < 0.05

DNA methylation is a key regulatory mechanism in CNS health and disease. To determine the possible contribution of de novo methylation in the function of glial Glu transporters, we examined the global change of 5-methylcytosine by DNMT3B increase in the human retina Müller glia cell line, MIO-M1. We have previously shown that global DNA methylation levels respond to Glu excitotoxic concentrations, and that under such conditions, DNMT3B expression increases [31]. Accordingly, DNMT3B exogenous transfection significantly enhances global 5-methylcytosine (5-mC) levels in MIO-M1 cells transfected with 2 µg of the DNMT3B plasmid. The methylated cytosines of genomic DNA were measured with a colorimetric assay based on an optimized antibody that allows high specificity to 5-mC, with no cross-reactivity to unmethylated cytosine and no or negligible cross-reactivity to hydroxymethylcytosine within the indicated concentration range of the sample DNA. As expected, a lower plasmid concentration (1.5 µg) increases DNA methylation levels only slightly. We also observe that the treatment with D-aspartate or L-Glu at excitotoxic concentrations (1 mM) increases the 5-mC content in the MIO-M1 genome (Fig. 2).

**Fig. 3:**
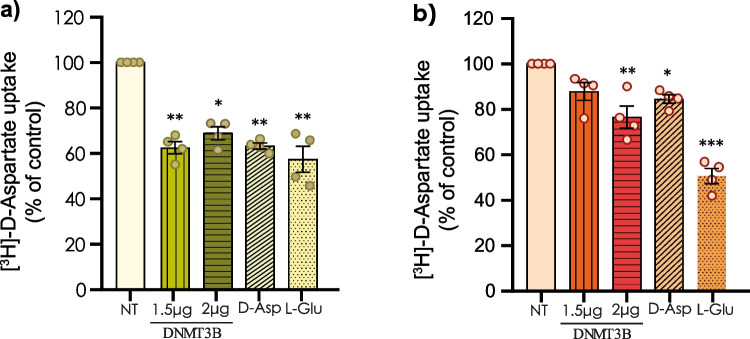
DNMT3B overexpression decreases the Glu uptake in glial cells. The immortalized human cells (**a**) MIO-M1 and the tumoral (**b**) U87 cells were transfected with DNMT3B vector by 48 h to evaluate the functional effect of DNA methylation increased in the [^3^H]-D-Aspartate uptake. Non-treated cells (NT) were used as reference to compare the uptake in both line cells transfected with 1.5 µg of DNMT3B plasmid (3B 1.5 µg) or 2 µg of DNMT3B vector (3B 2 µg). The positive controls for decreased uptake were measured in cells with 1 mM of D-Asp or 1 mM L-Glu treatments (D-Asp and L-Glu, respectively). The uptake of [^3^H]-D-aspartate (0.4 μCi/ml, for 30 min) was performed with a 50 µM D-Asp final concentration. In (**a**) MIO-M1 cells, all conditions decreased significantly the [.^3^H]-D-Aspartate uptake. The (**b**) U87 cells decreases significantly the uptake in control conditions (D-Asp and L-Glu) and upon methylating conditions with 2 µg of DNMT3B vector (3B 2 µg). Data is the average of three independent experiments performed in quadruplicates. Values represent mean ± SD. A one-way ANOVA was performed to determine whether there were significant differences between groups with a Dunnett test (Prism 9 software) *p < 0.05, **p < 0.01, ***p < 0.001

To address the plausible connection between DNA methylation and Glu transporter’s function, we performed [^3^H] D-Aspartate uptake assays under excitotoxic or hyper-methylating conditions. Since glia cells are severely disrupted in most of the neurodegenerative diseases and glioblastoma represents the highest cancer rates in the brain (associated with excitotoxic conditions), besides MIO-M1, we decided to use the U87 glioma cell model. The uptake of [^3^H]-D-Aspartate was reduced by DNMT3B overexpression with both plasmid concentrations in the two cellular models. Furthermore, under excitotoxic conditions (D-Asp and L-Glu treatments) glia cells present a reduction in [^3^H]-D-Aspartate uptake (Fig. 3a). The glioblastoma cell line, U87, had a similar behavior and took up less [^3^H]-D-Aspartate after D-Asp or GLU treatments, and after the higher DNMT3B plasmid (2 µg) concentration transfection, an uptake decrease is evidenced (Fig. 3b).

### EAAT1 and EAAT2 are Downregulated by DNMT3B Overexpression

**Fig. 4:**
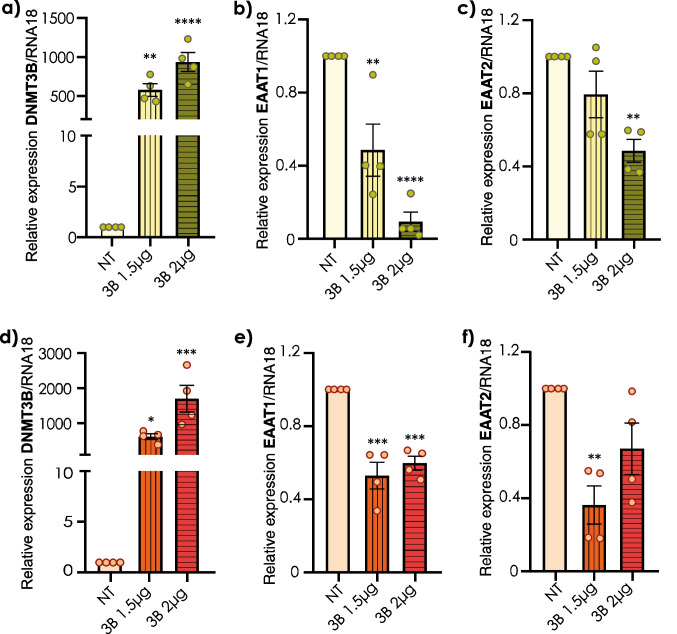
DNMT3B overexpression in glial cells results in the SLC1A2 and *SLC1A3* mRNA decrease. The immortalized human cells MIO-M1 cells and the tumoral U87 cells were transfected with 1.5 µg of DNMT3B plasmid (3B 1.5 µg) or 2 µg of DNMT3B vector (3B 2 µg) by 48 h. Total RNA was extracted in MIO-M1 and the relative expression was measured by RT-qPCR for (**a**) DNMT3B, **b** *SLC1A2* (EAAT2), and **c** *SLC1A3* (EAAT1). The Glu transporters decreased their expression upon DNMT3B overexpression in MIO-M1. The same experimental conditions were used for U87 cells and the (**d**) DNMT3B overexpression also has similar results, with an expression decrease in (**e**) *SLC1A2* and (**f**) *SLC1A3* mRNA. Each bar is the mean ± SD from 3 independent experiments by triplicates and normalized using *RNA18*. One-way ANOVA was performed to determine whether there were significant differences between groups with a Bonferroni test (Sigma Plot 12 Software). *p < 0.01; **p < 0.001; ***p < 0.0001, ****p < 0.00001

Previous studies indicate a high degree of post-transcriptional regulation of EAAT1/GLAST and EAAT2/GLT1, which involves, for instance, the Glu-dependent translocation process [36–39]. Nevertheless, a common additional regulation of *SLC1A2* and *SLC1A3* is elicited using alternative transcription starting sites (TSS) (Fig. S1) and several TF that regulate their transcriptional rates [26, 40–43]. To investigate if the decrease in the transporter’s function is related to changes in the transcriptional process that could be associated with the direct or upstream de novo DNA methylation process, we measured the mRNA levels of EAAT1 and EAAT2 after DNMT3B overexpression (Fig. 4a) in MIO-M1 cells, the expression of EAAT1 was reduced by the treatment with 1.5 µg and 2 µg of DNMT3B plasmid (Fig. 4b). In similar experimental conditions, we noted that the EAAT2 expression was decreased by the transfection of 2 µg DNMT3B plasmid (Fig. 4c). We performed the same evaluation for EAAT1 and EAAT2 mRNAs in the glioblastoma model (U87), and de novo DNA methyltransferase overexpression (Fig. 4d) results in a downregulation of both transporters' expression levels (Fig. 4e, f).

### De Novo DNA Methylation Targets the EAAT2 Promoter

**Fig. 5:**
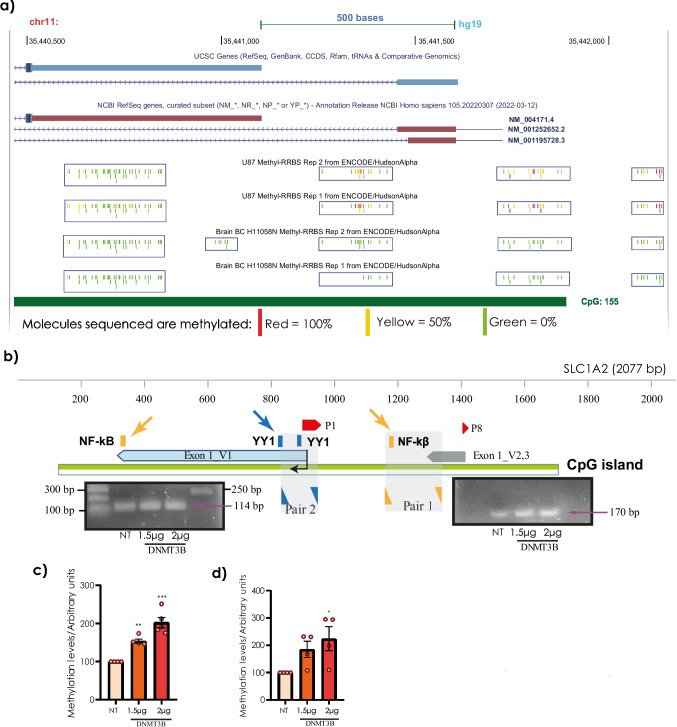
Enriched of DNA methylation on *SLC1A2* regulatory regions in glioblastoma cells is related to DNMT3B overexpression. **a** Screening in the genome browser shows high levels methylation on CpG located on the *SLC1A2* promoter region in U87 cells compared to normal brain tissues (Brain BC H11058N). The methylation changes (denoted in the box with red and yellow lines) were found near exon 1 and overlapping with the promoter region. **b** The U87 cells were transfected with 1.5 or 2 µg of DNMT3B plasmids and were evaluated by methylation-specific PCR (MSP) for two regions from *SLC1A2* CpG island (green box). In the first region (Pair 1, yellow) the binding site for NF-kβ (−251, arrows and yellow boxes) is found, and region 2 (Pair 2, blue) overlaps with the binding site for YY1 (+ 34, arrow and blue boxes). In red tag are indicated Promoter 1 (P1) that regulate the variant 1 of *SLC1A2* and the Promoter 8 (P8) that precede the exon 1 of variants 2 and 3. **c** and **d** Densitometric quantification of MSP products in agarose gels. The MSP assays were made at least 3 independent experiments by triplicates. Ctrl, transfected with plasmid control, 3b_1.5 µg and 3b_2µg, using DNMT3B plasmids in the concentration indicate. One-way ANOVA was performed to determine whether there were significant differences between groups with a Bonferroni test (Sigma Plot 12 Software). *p < 0.01; **p < 0.001; ***p < 0.0001

The *SLC1A2* (EAAT2 gene) structural characteristics contain key elements for DNA methylation deposition, such as a proper CpG island extending on the regulatory promoter region; in this element, we also found the transcription binding sites for YY1, NF-kB, CREB, and others (Fig. 5). It is well known that DNA methylation affects the binding of these transcription factors and regulates the transcription rates of target genes [44–48]. From the available information of the genome browser server, we obtain DNA methylation data of GLT1 in U87 and healthy brain tissue. By bisulfite-seq from the ENCODE project, the CpG methylation levels are higher in U87 cell respect to Brain BC H11058 upstream of the *SLC1A2* TSS (Fig. 5a). These high methylation levels in U87 cells are widespread on CpG located in the proximal (0–200 nt) and distant (> 300 nt) *SLC1A2* promoter that codifies the canonical EAAT2 isoform (NM_004171.4). The CpG island of *SLC1A2* has been reported to be methylated in glioma cell lines with respect to normal brain tissue [24]. To gain insight into the role of DNA methylation and the local regulation of EAAT2, we measured by MSP two regions in which we found binding sites for NF-kB (pair 1) and YY1 (pair 2). This qualitative assay shows an increased methylation on these regions when we overexpress de novo DNA methyltransferase DNMT3B (Fig. 5b).

## Discussion

Glial Glu transporters are the most important molecular machinery that maintain low extracellular L-Glu concentrations in the CNS [49]. Our current knowledge suggests that EAAT1 and EAAT2 levels are mainly regulated by posttranslational mechanisms, including membrane translocation or rapid protein degradation controlled by phosphorylation, ubiquitination, nitrosylation, and others [50–53]. Herein, we report a plausible transcriptional regulation by a DNA methylation process. *SLC1A2* and *SLC1A3* transcriptional regulation includes a strong stimulation by growth factors and other transcription factors that augments their mRNA levels [40]. Additionally, alternative splicing mechanisms have also been reported. In addition, other tracks of transcriptional regulation were included in this study. In silico analysis in the FANTOM5 SSTAR database (https://fantom.gsc.riken.jp/5/sstar/Main_Page) showed alternative transcription start sites in the *SLC1A2* and *SLC1A3* genes. These promoters have activity in several tissues, and the canonical promoter 1 presents tissue-dependent activity rates (Fig. S1). Another key factor for *SLC1A2* and *SLC1A3* local promoter regulation is epigenetic control by DNA methyltransferases and histone deacetylases (HDACs). Astrocytic EAAT1 and EAAT2 are diminished by the co-repression by YY1 and HDACs in rat primary cultures treated with manganese [25, 26]. Specifically in cultured astrocytes, TNF-alpha expression and Jun N-terminal kinase (JNK) phosphorylation result in an enhanced HDAC2 expression and consequent GLT-1 decrease [54]. Scarce information regarding the complexes that establish specific or dynamic DNA methylation patterns has been reported, although in human genomes, the interaction between HDACs and DNMTs has been identified, including DNMT3B to regulate 5mC deposition [55–57]. Studies in our laboratory suggest that a dynamic DNA methylation regulation of Glu transporters expression is present in specific excitotoxic conditions. Recently, it has been described that DNA methylation is a strong transcriptional regulator in the brain related to diseases. For example, DNA methylation in specific brain cell types could be used to map brain disorders [58], or *cell-free* DNA methylation emerges as a non-invasive biomarker for AD diagnosis [59]. We therefore investigated the status of *SLC1A2* and *SLC1A3* expressions in clinical samples from public databases and found a frequent deregulation in glial cell cancer and in some neurodegenerative diseases (Fig. 1). These findings suggest that a critical mechanism, like dynamic DNA methylation, is involved in a differential transcriptome that could contribute to these pathologies. To gain insight into this possibility, we used the human retina-derived Müller glia cell line, MIO-M1 cells. Accordingly, DNMT3B increases 5mC levels in a similar manner to the excitotoxic condition simulated by 1 mM of Glu or D-Aspartate (Fig. 2). In addition, the functional abilities for Glu clearance in the retinal cells (Fig. 3a) and in glioblastoma (Fig. 3b) cells decrease under hypermethylating conditions by the de novo DNA methylase, DNMT3B. These cell models are used as a preliminary approach, considering the heterogeneity and physiology of these cells, with the advantage that they are widely used models in CNS research. In neurodegenerative retinopathies, like *retinitis pigmentosa*, DNMT3A/3B are involved in the photoreceptor damage present in this illness. In line with this, it has been shown that the retinal damage induced by an alkylating agent is accompanied by DNMT3A/3B overexpression in a mouse model [60]. In glioma studies, active transcription marks on the DNMT3B promoter have also been found, and the use of DNMTs inhibitors resulted in loss of methylation and an increase in expression of target genes [61]. According to studies on excitotoxity, DNMTs are also increased directly by excitotoxic conditions, and the consequence is a global increase of DNA methylation in astrocytes co-cultured with neurons and endothelial cells [62]. DNA methylation has been described and studied due to its clear contribution to neurological disorders and neurodegenerative diseases, being a possible therapeutic (approach) target (review in [63, 64]). Demethylating treatments with pharmacological inhibitors of DNMTs are sufficient to protect neurons from Glu-triggered cell death [65]. In a similar neuronal landscape, both EAAT1 and EAAT2 expression are necessary for the tolerance of hiPSC-derived neurons to excitotoxicity [49]. With the compiled evidence published and, in this study, it is highly possible that an unexplored connection between DNA methylation and glial Glu transporters regulation in excitotoxic environments is central to neurological diseases in brain tissue.

Previous results in our work group and others demonstrate that DNMT3B expression is increased in response to excitotoxic Glu concentrations, and that DNMT3B overexpression in human glial cells downregulates EAAT1 and EAAT2 mRNA levels (Fig. 4). These findings suggest that transcriptional regulation through DNA methylation is a process that affects both glial transporters in a similar mechanistic manner directly in a differential dynamic methylation pattern of their promoters or the result of the alteration of intermediaries’ molecules (TFs, protein regulators) that regulate their expression. In this study, the data obtained from Glu uptake and *SLC1A3* expression assays indicate possible indirect regulation via DNMT3B, which needs to be explored using specialized methodologies to determine its nature. We decided to focus on GLT1 given the existing literature. Studies on GLT1, the rodent analog of EAAT2, demonstrated that DNA demethylation on selective CpG sites was highly correlated to increased mRNA levels in astrocytes from co-cultured with neuronal cells [66]. In glioma, it has been determined that the lack of EAAT2 expression is associated with a densely methylated *SLC1A2* promoter, in contrast with normal tissue, which is hypomethylated in the same promoter regions [24]. It is very important to note that DNA methylation acquires functional significance depending on the gene region and whether it occurs at key reading frames for proteins or transcription factors. It can be truly relevant even if it occurs at a single CpG site. In this regard, existing data on EAAT2 methylation has not focused on functional consequences. In this study, methylation was detected near two regions containing binding sites for key factors in the transcriptional regulation of this transporter. Based on the investigation support and the elements present in the SLC1A2 sequence, including CpG islands, we found a gain of methylation in the promoter region that overlaps with the CpG island in the U87 available data from methyl RRBS assays. This comparison was made with a brain non-tumoral cell line (Fig. 5a). In the same line, we also detected an increase of CpG methylation in two key sites for *SLC1A2* transcriptional regulation. Pair 1 of oligonucleotides surrounds the NFKβ binding site, a positive regulator of EAAT2, which can explain the expression decrease since methylated cytosine-content binding sequence changes the biophysical characteristics, and some transcription factors avoid the cognate DNA recognition [67]. Pair 2 enclosed a YY1 binding site, previously described as a negative regulator of EAAT2, that co-immunoprecipitated with the repressive complex PRC2. An additional mechanism of DNA methylation-mediated repression occurs by the Methyl-CpG-binding proteins and co-repressor complexes [68]. In this sense, it has been found to be a repressive chromatin complex that evidences the interaction between YY1/PRC2 [29, 69, 70], YY1/HDACs [28, 71], PRC2/DNMTs [72, 73], and DNMTs/HDACs [74, 75]. With this information, we can speculate that the methylation detected with Pair 2 could be a consequence of the interaction between YY1 and co-repressors. This hypothetical suggestion needs to be taken up in future studies.

Beyond the methylation of specific genes, the methylation of genes in groups (CpG Island Methylator Phenotype) stands out as an excellent clinical biomarker that has been explored in more than one type of cancer, highlighting the importance of methylation and dysregulation of DNMTs. In studies of heterogeneous gliomas, common IDH-mutants exist that are related to Glioma CpG Island Methylator Phenotype (G-CIMP). These phenotypes (G-CIMP-high and G-CIMP-low) are relevant clinically for predicting patient outcomes [76, 77].

**Fig. 6:**
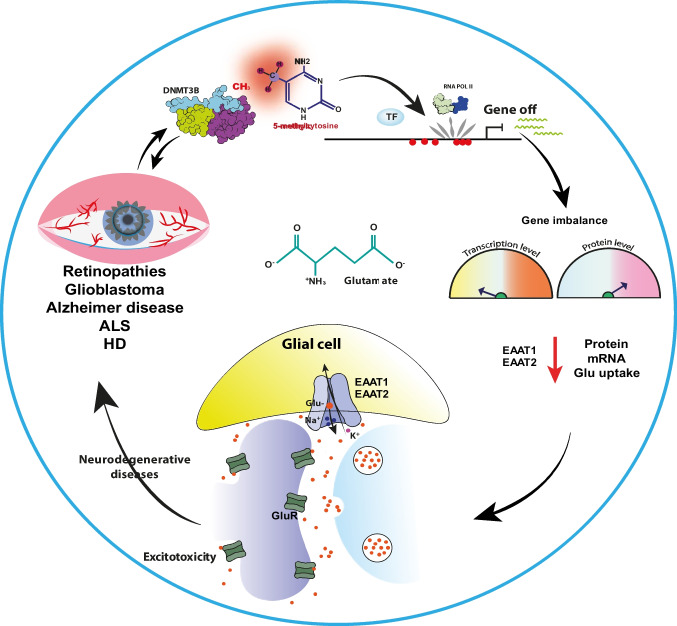
Proposed relationship between de novo DNA methylation, glial Glu transporters expression, and their contribution to neurodegenerative diseases (ND). DNMT3B is functional in CNS and regulated the transcription of several genes; its deregulation is a frequent result in several pathological conditions. De novo methylation deposition affects the transcription levels of key genes and contributes to the genesis or progression of ND. The DNMT3B overexpression decrease *SLC1A2* and *SLC1A3* mRNA levels and the functional consequence is the Glu uptake decrease in glial cells. The disruption of the correct Glu clearance by glial cells is a strong excitotoxic insult to neuronal cells in several ND, such as retinopathies, Parkinson disease (PD), Huntington disease (HD), Alzheimer disease (AD), glioblastoma, and others.

In conclusion, DNMT3B DNA methylation and the functional glial Glu transporters decrease could be a key axis for the deficient Glu clearance in pathophysiological conditions, in which DNMTs and EAATs are involved (Fig. 6). Despite these insights, further mechanistic studies are needed to discern the direct relationship of the *so-called* Excitotoxicity-DNMTs-SLC1A2 Methylation axis and to corroborate the specific DNA methylation pathway for *SLC1A2* transcriptional regulation. The involvement of methylation machinery, proteins, and other molecular elements involved in *SLC1A2* methylation needs to be investigated in future studies.

## Supplementary Information

Below is the link to the electronic supplementary material.

ESM 1 Supplementary Material 1 (AI 1.86 MB)

## Data Availability

All data generated or analyzed during this study are included in this published article.
